# Wood ants produce a potent antimicrobial agent by applying formic acid on tree‐collected resin

**DOI:** 10.1002/ece3.2834

**Published:** 2017-03-06

**Authors:** Timothée Brütsch, Geoffrey Jaffuel, Armelle Vallat, Ted C. J. Turlings, Michel Chapuisat

**Affiliations:** ^1^ Department of Ecology and EvolutionUniversity of LausanneBiophore, UNIL‐SorgeLausanneSwitzerland; ^2^ FARCEInstitute of BiologyUniversity of NeuchâtelNeuchâtelSwitzerland; ^3^ Institute of ChemistryNPACUniversity of NeuchâtelNeuchâtelSwitzerland

**Keywords:** antimicrobials, ants, chemical defenses, formic acid, *Formica*, fungal pathogen, social insects, tree resin

## Abstract

Wood ants fight pathogens by incorporating tree resin with antimicrobial properties into their nests. They also produce large quantities of formic acid in their venom gland, which they readily spray to defend or disinfect their nest. Mixing chemicals to produce powerful antibiotics is common practice in human medicine, yet evidence for the use of such “defensive cocktails” by animals remains scant. Here, we test the hypothesis that wood ants enhance the antifungal activity of tree resin by treating it with formic acid. In a series of experiments, we document that (i) tree resin had much higher inhibitory activity against the common entomopathogenic fungus *Metarhizium brunneum* after having been in contact with ants, while no such effect was detected for other nest materials; (ii) wood ants applied significant amounts of endogenous formic and succinic acid on resin and other nest materials; and (iii) the application of synthetic formic acid greatly increased the antifungal activity of resin, but had no such effect when applied to inert glass material. Together, these results demonstrate that wood ants obtain an effective protection against a detrimental microorganism by mixing endogenous and plant‐acquired chemical defenses. In conclusion, the ability to synergistically combine antimicrobial substances of diverse origins is not restricted to humans and may play an important role in insect societies.

## 1 Introduction

Animals living in large social groups are exposed to a high risk of epidemics. In response to this threat, social animals have evolved sophisticated individual and collective means to control disease, which combine immunological, behavioral, and organizational defenses (Cremer, Armitage, & Schmid‐Hempel, 2007; Naug & Smith, 2007; Wilson‐Rich, Spivak, Fefferman, & Starks, 2009). Collective defenses include ways to keep the environment hygienic, for example, by removing or neutralizing infectious particles (Morelos‐Juárez, Walker, Lopes, & Hughes, 2010; Tragust et al., 2013).

An original way to fight enemies is to exploit the defensive chemicals produced by other organisms (de Roode, Lefèvre, & Hunter, 2013). Humans use a myriad of chemicals from multiple sources, alone or in synergistic combinations, to medicate themselves, clean their environment, or control pests (Mason & Singer, 2015). Animals also harness chemicals produced by other species for their own defense (de Roode et al., 2013; Mason & Singer, 2015). For example, many insect herbivores sequester plant secondary metabolites to gain protection against predators or parasites (Lefèvre, Oliver, Hunter, & de Roode, 2010; Nishida, 2002). It has been proposed that animals may combine multiple acquired chemicals to benefit from synergistic effects (Mason & Singer, 2015). However, evidence for the use of such “defensive cocktails” by animals remains scant (Mason & Singer, 2015).

Wood ants and honeybees incorporate tree resin with antimicrobial properties into their nest (Christe, Oppliger, Bancala, Castella, & Chapuisat, 2003; Simone‐Finstrom & Spivak, 2010). In the wood ant *Formica paralugubris*, workers actively collect large amounts of resin from coniferous trees, which they bring back to their nest and place near their brood (Brütsch & Chapuisat, 2014; Castella, Christe, & Chapuisat, 2008). Coniferous resin is rich in secondary metabolites with antimicrobial properties (Phillips & Croteau, 1999). The presence of resin decreases the overall microbial load in wood ant nests and protects the ants against bacterial and fungal pathogens (Chapuisat, Oppliger, Magliano, & Christe, 2007; Christe et al., 2003).

Wood ants are also chemical factories. They produce large quantities of formic acid in their venom gland, which they spray at prey and enemies (Blum, 1992; Morgan, 2008). In other ant species, formic acid is also present in the trophallactic fluid following oral uptake from the venom gland (Tragust et al., 2013), and other acids have been found in the metapleural glands (Vieira, Morgan, Drijfhout, & Camargo‐Mathias, 2012). Formic acid has well‐known antimicrobial properties. It is widely used by humans, as cleaning agent and as preservative additive in livestock food (Iba & Berchieri, 2007). Moreover, formic acid is effective against *Metarhizium*, a common fungal pathogen of ants (Graystock & Hughes, 2011), and is used by *Lasiu*s *neglectus* ants to disinfect their brood (Tragust et al., 2013). This suggests that wood ants may combine endogenous acids with tree resin.

Here, we test the hypothesis that wood ants apply self‐produced acids on tree‐collected resin to produce a more potent antimicrobial agent. Specifically, we examined whether (i) ants enhance the antifungal activity of resin, (ii) ants add endogenous acids to resin, and (iii) these acids increase the antifungal activity of resin.

## 2 Materials and Methods

### 2.1 Effect of wood ants on the antifungal activity of resin

In a first experiment, we tested whether spruce tree resin that had been in contact with wood ants had increased inhibitory activity against the generalist entomopathogenic fungus *Metarhizium brunneum*, compared to resin that had not been contacted by ants. As controls, we used twigs and small stones. Twigs are major constituents of wood ant nests, and small stones are commonly found in some of the nests (Castella et al., 2008).

We established experimental groups of workers from field colonies of *Formica paralugubris* (Chapuisat, Goudet, & Keller, 1997; Christe et al., 2003). We collected pieces of fresh resin from spruce trees, as well as twigs and stones of similar size, in areas away from ant colonies. The pieces of resin, twigs, and stones were disinfected under UV light (30 mn under a UV lamp radiating at 254 nm in a Biosafety Cabinet BSC—700II, HMC Europe).

Each tested material (pieces of resin, twigs, and stones) was kept with and without ant workers for 2 weeks. In ant‐exposed treatments, four pieces of the tested material were kept with 40 workers in a small plastic box (13.5 × 15 × 5 cm; *n* = 25 replicates for each material). In ant‐free controls, four pieces of the tested material were kept in a box without workers (*n* = 25 replicates for each material). The edges of the boxes were treated with Fluon to prevent ant escape. The workers were free to interact with the pieces of resin, twigs, and stones. They had ad libitum access to water and jelly food consisting of chicken eggs, honey, water, and agar (Reber & Chapuisat, 2012a).

After this 2‐week period of exposure to ants or ant‐free control conditions, we measured the inhibitory activity of resin, twigs, and stones against the fungus *M. brunneum*. We used a strain that had been isolated from Valais, Switzerland, and showed high virulence against *Formica selysi* (Reber & Chapuisat, 2012b). *M. brunneum* was described in 2009 and was previously known as *M. anisopliae anisopliae* (Bischoff, Rehner, & Humber, 2009). A strain of the latter species complex caused high mortality to *F. paralugubris* (Chapuisat et al., 2007). *M. brunneum* is used here as a model fungal pathogen, while other pathogens might be important in the field. Indeed, the resin affects a broad spectrum of fungi and bacteria that are potential pathogens of *F. paralugubris* (Chapuisat et al., 2007; Christe et al., 2003).

Inhibitory activity was measured on Malt extract agar nutritive medium in 8.5‐cm‐diameter petri dishes, inoculated by plating 100 μl of 0.05% Tween 20 solution containing 7 × 10^5^ asexual spores (=conidia) of *M. brunneum*. The four pieces of each material (resin, twigs, or stone) coming from the same experimental box were placed together in a petri dish. The petri dishes (*n* = 25 per material and treatment) were incubated at 25°C for 6 days. We then photographed each petri dish and measured the spore‐free areas around the tested material with the *ImageJ* software (Schneider, Rasband, & Eliceiri, 2012). Spore‐free areas either were void of both spores and mycelium or consisted of white and mostly sterile mycelium known as sectors (Ryan, Bridge, Smith, & Jeffries, 2002).

For the statistical analysis, we used one estimate of inhibitory activity per experimental box (=replicate). We therefore measured the overall spore‐free area in each petri dish and divided it by four. This is a conservative estimate of the average inhibition halo around each item, because large halos were overlapping. We constructed a model with the spore‐free area as response variable, and the material (resin, twigs or stone) and previous contact with workers (presence or absence of workers in the box) as explanatory variables. The response variable was square‐root‐transformed to achieve homogeneity of variances and normal distribution of residuals, as required for an ANOVA. We carried out post hoc comparisons with Tukey's HSD tests.

### 2.2 Transfer of endogenous acids to resin and other types of nest material

In a second experiment, we examined whether ants applied endogenous acids to pieces of resin, twigs, or stones. We placed four pieces of the tested material (resin, twigs, or stone) in boxes with and without ants as described above, except that there were 50 workers per box in the treatment with ants (*n* = 10 replicates for each material and treatment type, with or without ants). As organic acids are very soluble in water, we sampled the acids from each material (resin, twigs, or stone) by immersing the four items from the same experimental box in 1 ml of MilliQ water for 30 s. The samples were stored at −20°C until HPLC analysis (see below).

We also tested whether the retention and subsequent detection of formic acid varied with the type of material (resin, twigs, and stone). For this, 1 μl of 60% synthetic formic acid (CAS number 64‐18‐6) was deposited on each type of material (10 replicates per material and treatment). After 24 hr, each item was immersed in 500 μl of MilliQ water for 30 s. The samples were stored at −20°C until HPLC analysis.

To identify the origin of the acids detected on nest materials, we extracted the content of the venom gland, trophallaxis fluid, and metapleural glands from ten workers anesthetized with CO_2_. For venom and trophallaxis fluid, we gently pressed their gaster and collected the liquid with a microcapillary. For the metapleural glands, we introduced the tip of a microcapillary in the gland opening and extracted the liquid by capillarity. We diluted these extracts in 500 μl of MilliQ water. The samples were stored at −20°C until HPLC analysis.

To measure the organic acids in the samples, we analyzed them by high‐performance liquid chromatography (HPLC), using an Agilent HP1100 HPLC system equipped with a diode array detector (DAD), with UV detection wavelength set at 210 ± 2 nm. To remove small particles, the samples were centrifuged (3 min at 182 g) and the supernatant was transferred to 2‐ml glass vials (Interchim, Swiss Labs, Mulhouse, F). We injected 40 μl of the samples onto a 300 mm × 7.8 mm BP‐100 H carbohydrate column (Benson Polymeric, USA). The temperature of the column was maintained at 40°C, and MilliQ water was used as a solvent with 20 mmol/L of sulfuric acid (analysis grade 95–97%, Honeywell, Germany) at a flow rate of 0.4 ml/min. Succinic (CAS number 150‐90‐3, Acros organics, USA) and formic (CAS number 141‐53‐7, Sigma Aldrich, USA) acids were quantified in the samples by external calibration. The linearity of the method was established using six standard solutions at concentration levels from 5 μg/mL to 1.3 mg/mL.

We constructed a model with acid quantity as response variable and the material (resin, twigs, or stone) and previous contact with workers (presence or absence of workers in the box) as explanatory variables. We analyzed the data of each acid separately.

### 2.3 Effects of acids on the antifungal activity of resin

In a third experiment, we tested whether combinations of synthetic acids corresponding to the composition of ant endogenous acids enhanced the antifungal activity of the resin. We mixed commercially available acids with MilliQ water to obtain a formic acid solution (formic acid 58.5%), a venom‐like mix (formic acid 58.5%, succinic acid 1.2%), and a trophallaxis‐like mix (succinic acid 3.6%, formic acid 0.06%) corresponding to the proportions of the main acids found in the venom and trophallactic fluid, respectively (see results). MilliQ water was used as control.

Pieces of spruce resin and pieces of safety glass were dipped in water, 58.5% formic acid, venom‐like or trophallaxis‐like mixes of synthetic acids, respectively. Safety glass was chosen as control because it is chemically inert. The amount of acid retained by pieces of glass and resin (after being dipped in acid) was not significantly different (0.011 ± 0.003 vs. 0.013 ± 0.006 g, respectively; *t *=* *−1.56, *df* = 42.83, *p *=* *.13; *N* = 30 pieces of each material). Inhibitory activity against *M. brunneum* was estimated by measuring the spore‐free area around each item. We used the procedure described above, except that we plated 250 μl of solution containing 4.5 × 10^6^ spores of *M. brunneum* on a nutritive medium of sabouraud glucose agar complemented with the antibiotics dodine, cycloheximide, and chloramphenicol, in 13.5‐cm‐diameter petri dishes, which allowed for better fungal growth. For each material, we placed one item subjected to each of the three acid treatments (dipped in 58.5% formic acid, venom‐like, and trophallaxis‐like mixes of synthetic acids) and to control conditions (dipped in water) on the same petri dish (*n* = 25 replicates per material).

For each material, we calculated the increase in antifungal activity due to exposure to acids. Specifically, we subtracted the spore‐free area produced by the control from the area produced by the acid‐exposed material in the same petri dish. We used Wilcoxon rank‐sum tests to examine whether the acid‐induced changes in antifungal activity differed between resin and inert glass material. All statistical analyses were performed in R version 3.3.0 (R Core Team 2016).

## 3 Results

### 3.1 Effect of ants on the antifungal activity of resin

**Figure 1:**
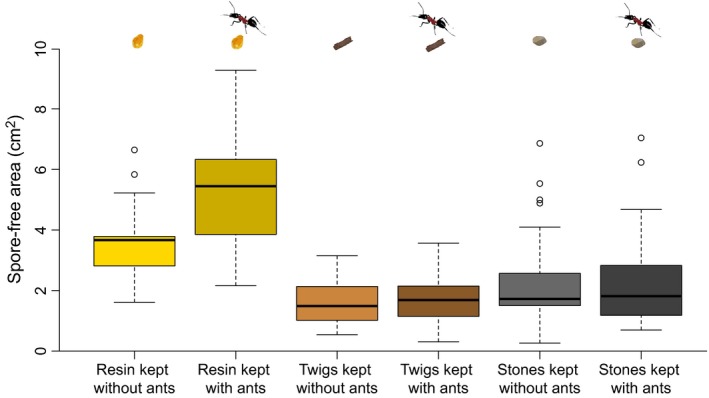
Antifungal activity of pieces of resin, twigs, and stones that had been kept without or with ants, respectively. The boxplots show the median values of spore‐free areas around the tested items, as well as the upper and lower quartiles. The whiskers encompass 1.5 times the interquartile range. Outliers are indicated by circles

Pieces of resin that had been kept with wood ant workers showed a significantly higher inhibitory activity against *M. brunneum* than pieces of resin that had not been in contact with ants. In contrast, the presence of workers had no effect on the antifungal activity of twigs and stones (Figure 1; ANOVA, interaction between material and contact with workers: *df* = 2, *F *=* *3.9, *p *=* *.022; Tukey's HSD post hoc tests: resin versus resin that had been in contact with workers: *p *<* *.0001; twigs versus twigs that had been in contact with workers: *p *=* *.99; stones versus stones that had been in contact with workers: *p *=* *.99). Overall, resin had higher antifungal activity than twigs or stones (Figure 1; ANOVA: *df* = 2, *F *=* *57.6, *p* < .0001).

### 3.2 Transfer of endogenous acids to resin and other types of nest material

Both formic and succinic acids were found on resin, twigs, and stones that had been in contact with workers (Table 1). In contrast, we did not detect these two acids on resin that had not been in contact with workers. We detected some succinic acid on twigs and formic acid on stones that had not been in contact with workers, but in much smaller quantities than on similar materials that had been in contact with workers (Table 1). Overall, we detected significantly more acids on materials that had been kept with ants (ANOVA, main effect of contact with workers: formic acid, *F *=* *34.8, *df* = 1, *p *<* *.0001; succinic acid, *F *=* *28.1, *df* = 1, *p *<* *.0001).

**Table 1:** Mean quantity of acids detected on resin, twigs, and stones that had been kept without ants or with ants, expressed as volume of acid in μl ± *SD*. The number of samples in which the acid was detected is given in parentheses (of 10 samples)

	Resin		Twigs		Stones	
	Kept without ants	Kept with ants	Kept without ants	Kept with ants	Kept without ants	Kept with ants
Formic acid	0 (0)	0.022 μl ±0.029 (10)	0 (0)	0.031 μl ±0.015 (10)	0.058 μl ±0.047 (9)	4.6 μl ±2.46 (10)
Succinic acid	0(0)	0.13 μl ±0.13 (10)	0.004 μl ±0.0094 (2)	0.049 μl ±0.031 (10)	0 (0)	0.097 μl ±0.083 (10)

For formic acid, there was a significant interaction between material and contact with ants (Table 1; *F *=* *33.6, *df* = 2, *p *<* *.0001). The high amount of formic acid detected on stones that had been kept with ants can be explained by large differences among the three materials in their ability to sequester and release formic acid. Indeed, when we experimentally deposited a controlled amount of 1 μl of 60% formic acid on each type of material, we detected much more acid on stones than on resin and twigs, respectively (mean in μl ± *SD*: stones, 0.41 ± 0.11; resin, 0.00044 ± 0.0014; twigs, 0.013 ± 0.0043; Kruskal–Wallis rank‐sum test: χ^2^ = 26.5, *df* = 2, *p *<* *.0001).

Worker ants produced large quantities of formic acid and comparatively small amounts of succinic acid. The venom gland extracts contained on average 58.5% of formic acid (detected in all 10 samples) and 1.2% of succinic acid (five samples). The trophallactic fluid contained 3.6% of succinic acid (nine samples) and 0.06% of formic acid (one sample). Fumaric acid was detected in trace quantities in the venom and trophallactic fluid. We did not detect any acid in the metapleural gland extracts.

### 3.3 Effects of acids on the antifungal activity of resin

**Figure 2:**
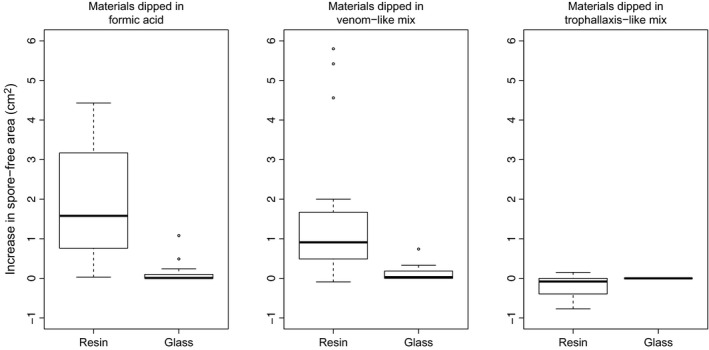
Increase in the antifungal activity of resin and glass dipped in 58.5% formic acid, venom‐like, and trophallaxis‐like mixes of synthetic acids, respectively, relative to controls (same materials dipped in water)

The treatment with synthetic formic acid at a concentration corresponding to the one of venom increased the inhibitory activity of resin against *M. brunneum* (Figure 2). Formic acid had a significantly stronger impact on the antifungal activity of resin than of inert glass material, which is indicative of a synergistic interaction (Figure 2; Wilcoxon rank‐sum test: *W *=* *323, *p *<* *.0001). The treatment with the venom‐like mix (formic + succinic acids) also increased the antifungal activity of resin, but not more than formic acid alone, at the same concentration (Figure 2). The increase in antifungal activity due to the venom‐like mix was also stronger for resin than for glass (*W *=* *312, *p *<* *.0001). The treatment with the trophallaxis‐like mix, which contains more succinic acid and only traces of formic acid, slightly decreased the antifungal activity of resin and had no effect on the antifungal activity of glass (Figure 2; *W *=* *70, *p *=* *.0009).

## 4 Discussion

Wood ants are known to incorporate plant resin with antiseptic properties into their nests (Christe et al., 2003; Simone‐Finstrom & Spivak, 2010). Here, we show that wood ants enhance the antifungal activity of tree‐collected resin by supplementing it with endogenous formic acid. Three lines of experimental evidence support this claim. First, tree resin showed significantly higher inhibitory activity against the fungal pathogen *M. brunneum* after having been in contact with wood ants. In sharp contrast, the contact with ants did not affect the antifungal activity of control materials commonly found in wood ant nests, namely twigs and small stones.

Second, the ants applied significant amounts of endogenous formic and succinic acid on resin and other types of nest material. The proportion of formic acid and succinic acid varied with substrate, which likely reflects differences among substrates in their ability to take up and release formic acid (Al‐Hosney, Carlos‐Cuellar, Baltrusaitis, & Grassian, 2005). Large quantities of formic acid and small amounts of succinic acid were found in the venom of wood ants.

Third, the treatment of resin with synthetic formic acid greatly increased the antifungal activity of the resin, but had no such effect on pieces of glass. This interaction between formic acid and substrate reveals a synergistic effect. Indeed, the combination of formic acid and resin produced a higher antifungal activity than the additive effect of each compound. The application of formic acid on resin was sufficient to obtain this synergistic effect, and succinic acid did not appear to contribute to the antifungal activity of resin. Together, these results provide strong evidence that wood ants apply formic acid produced in their venom gland on tree resin, which results in a synergistic increase in the antifungal activity of resin.

Documented cases of “defensive mixology,” whereby animals actively combine antimicrobial substances of diverse sources to obtain a synergistic protection, are extremely rare (Mason & Singer, 2015). Honeybee workers manipulate tree resin with their mandibles, but there is no evidence that this process modifies the chemical composition of the resin (Simone‐Finstrom & Spivak, 2010). Stingless bees collect resin from several plant genera. Although these resins vary in their antibacterial properties, mixing them had no synergistic effect against a fungus and various bacteria (Drescher, Wallace, Katouli, Massaro, & Leonhardt, 2014). Synergistic defenses may also occur in herbivores or nectar‐feeding animals (Mason & Singer, 2015). For example, a dietary treatment with a mix of thymol and nicotine tended to reduce the load of a protozoan parasite in bumblebees (Biller, Adler, Irwin, McAllister, & Palmer‐Young, 2015).

Like humans, social insects have extraordinary capacities to exploit and modify their environment (Wilson, 1971), and they rely on sophisticated means to keep pathogens at bay (Cremer et al., 2007). Here, we provide evidence that wood ants do not only collect tree resin with antimicrobial properties; they also supplement it with formic acid. Thus, wood ants combine their own chemical defenses with the ones of plants to produce a more potent antimicrobial agent that contributes to nest hygiene (Brütsch & Chapuisat, 2014; Castella et al., 2008; Christe et al., 2003) and protects the ants against detrimental microorganisms (Chapuisat et al., 2007). Together, these findings demonstrate that the ability to synergistically combine antimicrobial substances of diverse origins is not restricted to humans and may play an important role in insect societies.

## Author Contributions

MC conceived of the project. TB, GJ, and MC designed the experiments. TB and GJ carried out the experiments and analyzed the data. GJ and AV carried out the chemical analyses. TCJT contributed to data analysis and interpretation. TB and MC led the writing of the manuscript. All authors contributed critically to the drafts and gave final approval for publication.

## Conflict of Interests

The authors declare that they have no competing interests.

## Data Accessibility

All supporting data will be deposited in the public archive Dryad.
